# Preparation and Luminescence Properties of Ba_5_Si_8_O_21_ Long Persistent Phosphors Doped with Rare-Earth Elements

**DOI:** 10.3390/ma12010183

**Published:** 2019-01-07

**Authors:** Andrea Silvestri, Maria Laura Ligabue, Gianluca Malavasi, Gigliola Lusvardi

**Affiliations:** Department of Chemical and Geological Sciences, University of Modena and Reggio Emilia, Via G. Campi 103, 41125 Modena, Italy; 191646@studenti.unimore.it (A.S.); mlauraligabue@gmail.com (M.L.L.); gianluca.malavasi@unimore.it (G.M.)

**Keywords:** barium silicates, phosphors, kinetic analysis, quantitative phase analysis, luminescence

## Abstract

The phosphors of formula Ba_5_Si_8_O_21_:Eu^2+^,Dy^3+^ were synthesized and studied in order to improve their properties. Their synthesis conditions were evaluated as a function of precursors, crucible composition, flux agents, dopants and temperatures. The samples were characterised by means of a systematic investigation through elemental, kinetic, mineralogical (both qualitative and quantitative), and morphological analysis. This study allows for a careful evaluation of the parameters that influence the formation and properties of Ba_5_Si_8_O_21_:Eu^2+^,Dy^3+^ phosphors. As for the synthesis conditions, the use of Na_2_SiO_3_, BaCO_3_ and NH_4_Cl as precursors was very important to reduce the temperature and time of synthesis. The reducing atmosphere produced with purified coal was cheaper and gave results similar to the more traditional gas mixture (H_2_/N_2_). At the end of this study, a phosphor with improved long persistent phosphorescence (LPP) characteristics was obtained with Ba/Si = 0.7, Eu/Si = 2.8 × 10^−3^ and Dy/Si = 3.6 × 10^−3^ following a 6 h-synthesis in a quartz crucible.

## 1. Introduction

The term luminescence indicates the phenomenon of light emission from a material after the excitation of its electronic states by an external source [1,2]. Photoluminescence is the most widely occurring phenomenon, and it involves excitation by electromagnetic radiation. Depending on the material, photons can be emitted using the mechanism of fluorescence (light emission for less than 10^−8^ s) or phosphorescence (light emission for minutes or hours).

The materials which possess these characteristics are mostly inorganic compounds and are generally called phosphors [2,3,4]. 

In the last 30 years, other types of long persistent phosphorescence (LPP) [1,2,3,4,5,6,7] phosphors based on either alkali or alkaline-earth metal aluminates doped with rare earth ions or transition metals ions attracted much attention and were actively investigated [8]. In particular, special attention was given to strontium aluminates doped with Eu^2+^ and Dy^3+^, SrAl_2_O_4_:Eu^2+^/Dy^3+^ [9] and Sr_4_Al_14_O_25_:Eu^2+^/Dy^3+^ [10,11], which are characterised by a strong emission, centred in the range of green-blue at 520 and 495 nm, respectively, and a phosphorescence that lasts overnight. These compounds possess partial solubility in water [12] and, consequently, require a protective coating [13,14,15]. Therefore, their limited outdoor applications, possible increase in production costs and the challenging employment of their emission colour led to a further search for different phosphors.

Alkaline-earth silicate phosphors, synthesized in the last few years [16,17,18,19,20], have garnered much attention for their better physico-chemical properties [21,22], their stability over time and their lower synthesis temperature compared to those of aluminate-derived phosphors [18]. Moreover, these phosphors emit a wider range of light colours, like green [23], red [24], yellow [16] and white [25]. Unfortunately, the duration of their emission is lower compared to that of aluminate-derived phosphors; consequently, a lot of studies are aimed at improving this property. Yu Gong et al. [17] reported the synthesis of Ba_4_(Si_3_O_8_)_2_:Eu^2+^,Dy^3+^ as an LPP phosphor, with high chemical stability and an emission of more than 24 h after light excitation at λ = 500–550 nm. Later, Pengjiu Wang et al. [18] reported the preparation of Ba_5_Si_8_O_21_:Eu^2+^,Dy^3+^, that got a better luminescence characteristic due to its crystalline structure. This phosphor possesses sustained phosphorescence when activated by sunlight (λ = 473 nm), with a lasting time beyond 16 h. 

The LPP phosphors are studied by different researchers and their applications have increased from the civil uses (i.e., traditional displays, lighting, medicine, security) to a wide range of scientific fields, such as life sciences, biomedicine, clinical medicine, energy and environmental engineering [26,27,28].

In the present study, we aimed to further improve the synthesis of Ba_5_Si_8_O_21_:Eu^2+^,Dy^3+^ phosphors and theirs LPP characteristics. 

On the basis of our previous experience [29], we carried out a careful evaluation of the effect of precursors, crucible composition, flux agents, dopants, time and temperatures used in the synthesis. The characterization through elemental, kinetic, mineralogical and morphological analysis will help establish the best experimental conditions to obtain this kind of phosphors with improved properties.

## 2. Materials and Methods 

### 2.1. Synthesis

Barium silicate phosphors doped with Eu^2+^ and Dy^3+^ (Ba_5_Si_8_O_21_:Eu^2+^/Dy^3+^) were prepared through a solid-state reaction. The raw materials used consisted of barium carbonate (BaCO_3_; Riedel-de Haën, Hannover, Germany, 99.0%) or barium chloride (BaCl_2_; Carlo Erba, Cornaredo (MI), Italy, 99%), sodium metasilicate pentahydrate (Na_2_SiO_3_·5H_2_O; Aldrich, St. Louis, MO, USA, 95.0%) or silicon oxide (SiO_2_; Aldrich, purum), dysprosium oxide (Dy_2_O_3_; Aldrich, 99.9%), europium oxide (Eu_2_O_3_; Aldrich, 99.9%), ammonium chloride (NH_4_Cl; Riedel-de Haën, 99.5%) and boric acid (H_3_BO_3_; Aldrich 99.8%). Some phosphors (Ba_5_Si_8_O_21_:Eu^2+^/Er^3+^) were also doped with Er^3+^ (Er_2_O_3_; Aldrich, 99.9%).

The reagents were weighed with an analytical balance (±0.01 mg) according to the stoichiometric composition of Ba_5_Si_8_O_21_ and then mixed in an agate mortar; the mixture was dried at 100 °C for 2 h and successively put into a crucible. The reducing atmosphere was created with purified coal, which produces CO_2_ and CO on burning; it is, in fact, well-known that this kind of atmosphere is cheaper and more efficient for the sintering process than the one obtained with a more traditional gas mixture (H_2_/N_2_) [30]. The crucible with the mixture surrounded by coal was inserted inside a large alumina crucible, which was closed with a lid. The synthesis was carried out at 1100–1250 °C for 3–12 h, and the obtained material was then grounded in an agate mortar to obtain a fine powder used for the characterisation.

### 2.2. Characterisation

Mineralogical studies (phase identification and quantification) performed by XRPD (X-Ray Powder Diffraction) were carried out with a PANalytical X’Pert Pro Bragg-Brentano diffractometer (Panalytical, Malvern, UK), using Ni-filtered Cu Kα radiation (λ = 1.54060 Å) with an X’Celerator detector. The patterns were taken over the diffraction angle range of 2θ = 5–55°, with a time step of 50 s and a step size of 0.03° (angular step). In the case of quantitative phase analysis (QPA), the patterns were collected in the range of 2θ = 3–100°, with a time step of 100 s and a step size of 0.03°. The QPA results were elaborated by means of the combined Rietveld-reference intensity ratio (RIR) method [31]. QPA refinements of the powder spectra were performed using the GSAS software [32], and its graphical interface EXPGUI [33]. The structural models for all phases were taken from the ICSD database [34]. The refined instrumental parameters were the Chebyshev polynomial background function and the zero-shift. For each phase, the refined parameters consisted of the scale factor, unit-cell parameters, Gaussian and Lorentzian coefficients of the pseudo-Voigt peak-profile function, offset function for the correction of the peak asymmetry and sample-displacement correction.

Surface morphology and its composition were examined with a Scanning Electron Microscope (FEI Quanta 200, FeiCo., Abingdon, UK), equipped with an energy dispersive spectroscopy (EDS) instrument (INCA 350, Oxford Instrument, Abingdon, UK). EDS analyses were performed in quadruplicate for each examined agglomerate onto the surface; the result was a mean of the replicates and a standard deviation of 0.5%.

A quantification of Si and Ba was performed with an ICP spectrometer (Perkin Elmer Optima 4200 DV, Waltham, MA, USA), while for Eu, Dy and Er, an ICP-MS spectrometer was applied (X Series, Thermo Fisher Scientific, Waltham, MA, USA). The standard deviation for ICP results was lower than 1%, and the detection limit for ICP-MS shows was 0.05 pg/mL.

The afterglow decay was used to measure the luminous intensity with a luminance meter (Minolta CS-100A, Ramsey, NJ, USA). Following the procedure of P. Wang et al. [18], we excited each sample for 10 min with a Wood lamp (366 nm), after which the luminance was measured in a dark room (1 m was the distance between the sample and the Luminance Meter). The reproducibility determined was 0.005 cd/m^2^. The instrument works in the range of 0.002–49.900 cd/m^2^ with a sampling time of 0.4 s. The obtained decay curves were elaborated by means of a kinetic analysis with first, second and third-order functions. 

The best fitting was evaluated by the calculation of deviation (D%), which is defined as the root mean square offset between the experimental and computed data through the use of first, second, or third-order decay equations [35]. In other words, these results were compared since the conditions used in the tests are always the same.

In our samples, the afterglow decay can be fitted with a second-order function:

*y*(*t*) = *y*_0_ + *A*_1_*e*(−*t*/*τ*_1_) + *A*_2_*e*(−*t*/*τ*_2_)

where *y*(*t*) is the luminance emission intensity at time *t* after switching off the excitation source, *y*_0_ is the luminance emission at time zero, *A_i_* is a time-invariant constant that represents the amplitude of luminescence intensity corresponding to the *i* decay component, while *t_i_* is the corresponding decay time-constant. We used the values of *τ*_1_ and *τ*_2_ to compare the behaviour of the differently synthesized phosphor; it was possible to use *τ_m_* for this purpose. 

## 3. Results

We started from a reference phosphor Ba_5_Si_8_O_21_:Eu^2+^,Dy^3+^ [18], with these molar ratios: Ba/Si = 0.625, Eu/Si = 2.5 × 10^−3^ and Dy/Si = 11.25 × 10^−3^. These were obtained from BaCO_3_, SiO_2_, Dy_2_O_3_, Eu_2_O_3_ and H_3_BO_3_ (2.5 wt.%) by treatment at 1250 °C for 10 h in a platinum crucible. 

In order to perform an accurate comparison, we reproduced the synthesis of this phosphor in our laboratory and the product was named sample 1.

The sample synthesized with the same molar ratios and reagents and at the same annealing conditions but in a quartz crucible instead of platinum was named sample 2. Mineralogical studies confirm that in both cases, it is possible to obtain Ba_5_Si_8_O_21_ as the dominant crystalline phase and the sample 1 maintains their long-persistent phosphorescence characteristics. Then, the synthesis of this phosphor was evaluated by varying the types and amounts of precursors, dopants, flux agents, crucibles type, temperature and time of annealing. For all samples, the reducing atmosphere was created with purified coal.

Each sample was obtained by changing the variables step by step; this will be discussed in the following paragraphs.

In Table 1, the compositions and synthesis conditions of the samples (A–H) are reported with respect to those of sample 1.

### 3.1. Effect of the Precursors

Our first aim was to find precursors of silicon and barium that could react faster and at lower temperatures in contrast to those used for sample 1. The combined use of Na_2_SiO_3_ and BaCO_3_ as sources of silicon and barium produces a glassy compound instead of a crystalline one, probably due to the low fusion temperature of Na_2_SiO_3_ (T = 1088 °C). To avoid this problem and help the formation of a crystalline phase, we decided to use H_2_SiO_3_ instead of Na_2_SiO_3_ [17]. H_2_SiO_3_ was obtained by reaction between NH_4_Cl and Na_2_SiO_3_:

Na_2_SiO_3_ + 2NH_4_Cl → H_2_SiO_3_ + 2NH_3_ (↑) + 2NaCl

To confirm the correctness of this procedure, we carried out a mineralogical study [36] on the mixture with all precursors (BaCO_3_, Na_2_SiO_3_, NH_4_Cl, Eu_2_O_3_ and Dy_2_O_3_) before the heat treatment, and the results (Table S1, Supplementary Materials) indicated the presence of NaCl, BaCO_3_ and the absence of any foreign crystalline phase. Furthermore, the presence of NaCl is also useful as a flux agent for the synthesis.

**Figure 1:**
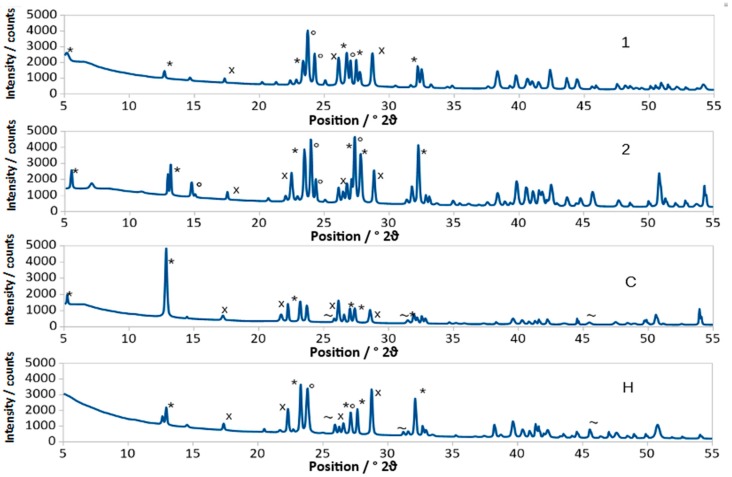
XRPD of samples 1, 2, C, H; Ba_5_Si_8_O_21_ [36, PDF350766] (*), Ba_4_Si_6_O_16_ [36, PDF831482] (°) BaSi_2_O_5_ [36, PDF260176] (x), NaCl [36, PDF882300] (~).

We have tried other reagents with the following aims:

- to reduce reagents number: using BaCl_2_ instead of NH_4_Cl and BaCO_3_. The mineralogical results indicate that even at high temperature (1200 °C) and for a long time (12 h), the final product showed the presence of some residual reagents. The reaction was not complete in terms of the formation of Ba_5_Si_8_O_21_ and displayed a weak afterglow luminescence with respect to the reference (Figure S1, Supplementary Materials).
- to use a low expensive reagent: using SiO_2_ instead of Na_2_SiO_3_, and H_3_BO_3_ as a flux agent instead of NaCl. The afterglow was good, but the reaction required a high temperature (1250 °C) and a long synthesis time (10 h) (Figure 1, sample 2).

Consequently, we decided to always use H_2_SiO_3_ (from NH_4_Cl and Na_2_SiO_3_) and BaCO_3_ as sources of silicon and barium, respectively. 

### 3.2. Effect of Crucible and Ba/Si Molar Ratio

In this case, our aim was to find cheaper and more versatile crucible materials and replace platinum. We tried crucibles constituting of different materials: platinum, alumina, boron nitride and quartz. We decided to discard platinum because of the lack of reproducibility of the photoluminescent properties, which is probably due to chemical interactions between the crucible and its reagents. Similarly, for the alumina crucible, the precursors interacted with the crucible and, in fact, from mineralogical analysis, the presence of a barium aluminate phase (Ba_2_Al_2_O_5_) was identified (Figure S2, Supplementary Materials). Boron nitride also interacts with the reagents, making it difficult to remove the mixture from the crucible.

Generally, in the case of the quartz crucible, the mineralogical analysis indicates the formation of competitive crystalline phases for the formation of Ba_5_Si_8_O_21_; these phases, BaSi_2_O_5_ and Ba_4_Si_6_O_16,_ are in agreement with the earlier reported phase diagrams of the system BaO-SiO_2_ [37]. In particular, the presence of BaSi_2_O_5_ and its Ba/Si molar ratio of 0.5, indicate a probable interaction with the quartz crucible and a consequent increase of the amount of Si inside the phosphor. To obtain the optimal Ba/Si molar ratio required for the formation of Ba_5_Si_8_O_21_, we increased the amount of BaCO_3_. Different Ba/Si molar ratios were tested (0.65, 0.66, 0.7, 0.75 and 1), and the most promising results was 0.7.

Consequently, we decided to use the crucible of quartz in all the samples. 

### 3.3. Effect of Flux Agents and of Eu/Si, Dy/Si Molar Ratios

On the basis of the previous considerations, the interesting results were obtained with a molar ratio Ba/Si = 0.7 with H_2_SiO_3_ and BaCO_3_ as precursors, synthesized in a quartz crucible. Starting from these parameters, we prepared a sample with the same type and amount of flux agent as in sample 1 (sample A), and sample B was prepared with NaCl (10 wt.%) instead of H_3_BO_3_ (2.5 wt.%). NaCl was obtained by the reaction of NH_4_Cl and Na_2_SiO_3_ (see Section 3.1). 

In sample B, the principal phase was Ba_5_Si_8_O_21_ (45 wt.%) and the secondary phases were Ba_4_Si_6_O_16_ (15 wt.%) and BaSi_2_O_5_ (10 wt.%). Contrary to that, in sample A, the principal phase was Ba_4_Si_6_O_16_ (44 wt.%), while Ba_5_Si_8_O_21_ (20 wt.%) and BaSi_2_O_5_ (15 wt.%) were the secondary ones. The H_3_BO_3_ use (sample A) lead to the dominant formation of Ba_4_Si_6_O_16_, which reduced the afterglow luminescence. In fact, the initial emission of sample B (*A*_1_ = 109 mcd/m^2^) was higher than that of A (*A*_1_ = 93 mcd/m^2^) (Table 2). 

To improve the reaction yield, different amounts of H_3_BO_3_ and dopant molar ratios were tested (samples C, D). Samples C and D have the same molar ratios and were synthesized at the same conditions, but they differ by flux agents and contain, respectively, NaCl and H_3_BO_3_ (10 wt.%).

Mineralogical analysis (Table S2, Supplementary Materials) allowed us to identify different crystalline phases. Due to pattern complexity (Figure 1), a qualitative study that refers to the diffraction peaks intensity is not sufficient to quantify the amount of each phase even from a semi-quantitative point of view. In fact, sometimes, there are peaks positioned very close to each other or overlapped (e.g., d = 3.73 Å and d = 3.74 Å respectively for Ba_5_Si_8_O_21_ and Ba_4_Si_6_O_16_) and, also, preferential orientation can appear (d = 6.88 Å for Ba_5_Si_8_O_21_).

Consequently, using the Rietveld method in order to perform a quantitative phase analysis (QPA) gives us more accurate results (Table 3); it indicated whether Ba_5_Si_8_O_21_ is the principal crystalline phase and also demonstrated other competitive crystalline phases besides the amorphous phase.

Ba_5_Si_8_O_21_ is the principal crystalline phase in both cases, with 59 wt.% for sample C and 76 wt.% for sample D. BaSi_2_O_5_ is a secondary phase presented at 13 and 7 wt.%, respectively, for samples C and D. Sample C also contains Ba_4_Si_6_O_16_ (8 wt.%).

**Figure 2:**
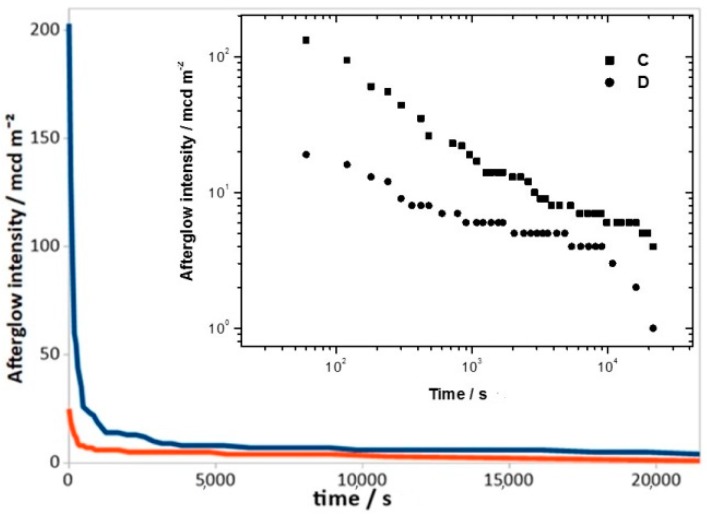
Decay curves of samples C (blue) and D (red). The upper inset showed the log-log plot.

The lower number of competitive phases in sample D makes it possible to suppose that H_3_BO_3_ could be the best flux agent. However, the luminescence decay curves (Figure 2) and the initial emission (*A*_1_) (Table 2) demonstrates that sample C has a better performance than sample D. Comparing with samples 1 and 2, we observe a higher value of *A*_1_ for C: 180 mcd/m^2^ for C, compared with 133 and 94 mcd/m^2^, respectively, for 1 and 2.

Therefore, we can suppose that H_3_BO_3_ (used in the sample D, 1 and 2) somehow inhibits the emission intensity in the samples.

We also prepared a sample with both flux agents, but the final result was an amorphous compound that did not show any phosphorescence.

These results indicate that, in our conditions, NaCl is the best flux agent.

For all studied samples, similarly to Y. Gong et al. [17] and P. Wang et al. [18], we used Eu^2+^ as emission source and Dy^3+^ to enhance the afterglow emission; different molar ratios (Eu/Si, Dy/Si) were tested, and the most promising ones are reported in Table 1. 

Furthermore, as seen from P. Wang et al. [38], we tried using Er^3+^ instead of Dy^3+^. 

We compared the luminescence emission and kinetic analysis of sample C (doped with Eu^2+^, Dy^3+^) and sample E (doped with Eu^2+^, Er^3+^) (Table 2). The initial emission *A*_1_ is higher for sample E: 213 against 180 mcd/m^2^. Unfortunately though, it has got a very short afterglow decay: *τ*_2_ = 494 s compared to *τ*_2_ = 6358 s of sample C.

We synthesised the samples with both Er^3+^ and Dy^3+^; the elements were added in the same molar ratios X/Si (X = Er or Dy) = 2 × 10^−3^, 3.5 × 10^−3^ and 3.6 × 10^−3^. In all cases, the initial emission was not comparable with sample E, while *τ*_2_ remained similar to one of the samples with only Eu^2+^ and Dy^3+^. Consequently, we decided to use only Eu^2+^ and Dy^3+^ as dopants.

The parameters reported in Table 2 indicated that the contribution to the total photon emission of the fast decay *A*_1_ × *τ*_1_ (%) is lower, as compared to the slow decay component *A*_2_ × *τ*_2_ (%). The fast and slow decay components correspond to 10 and 90%, respectively. This suggests that, for the major part of the phosphors, the afterglow decay process is the same, except for sample E (doped with Er^3+^), which has the brightest initial emission, but with the shortest afterglow time.

### 3.4. Effect of Heat Treatment

The synthesis conditions, in addition to raw material selection, play a crucial role in the process. Higher temperatures or longer thermal treatment times improved both the solid-state reaction and the formation of the desired crystalline phase responsible for photoluminescence. 

The most promising synthesis was carried out at 1100 °C, instead of 1250 °C used for the reference sample 1.

We tested different synthesis times: 12 h for sample F, 6 h for sample C, 4 h for sample G and 3 h for sample H. 

From QPA (Table 3), we can see that the principal phase is Ba_5_Si_8_O_21_ in all the samples. 

Sample G got the higher amount (66 wt.%) of Ba_5_Si_8_O_21_, but sample C (59 wt.%) possesses better phosphorescence, as one can see from the luminescence emission and kinetic analysis results (Table 2). 

**Figure 3:**
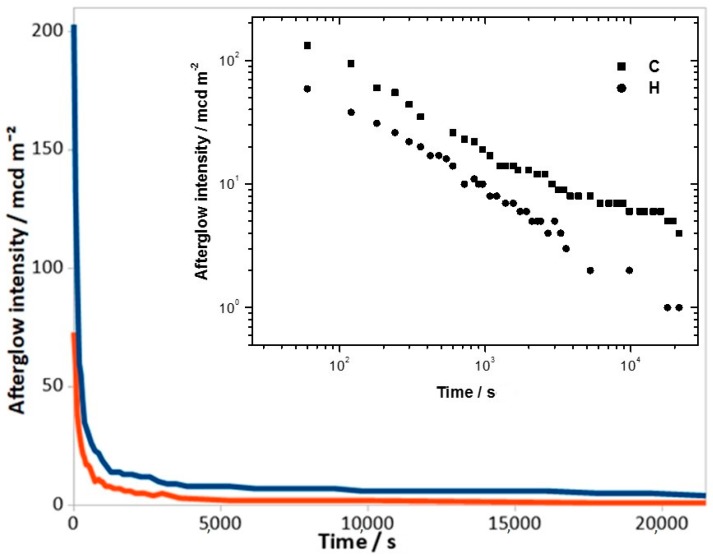
Decay curves of samples C (blue) and H (red). The upper inset showed the log-log plot.

The secondary phases amounts are similar in samples F, G and H. Sample H presents the greatest quantity of amorphous phase (27 wt.%), and this could explain the worst values in the luminescence decay curves and kinetic parameters (Figure 3, Table 2). 

**Figure 4:**
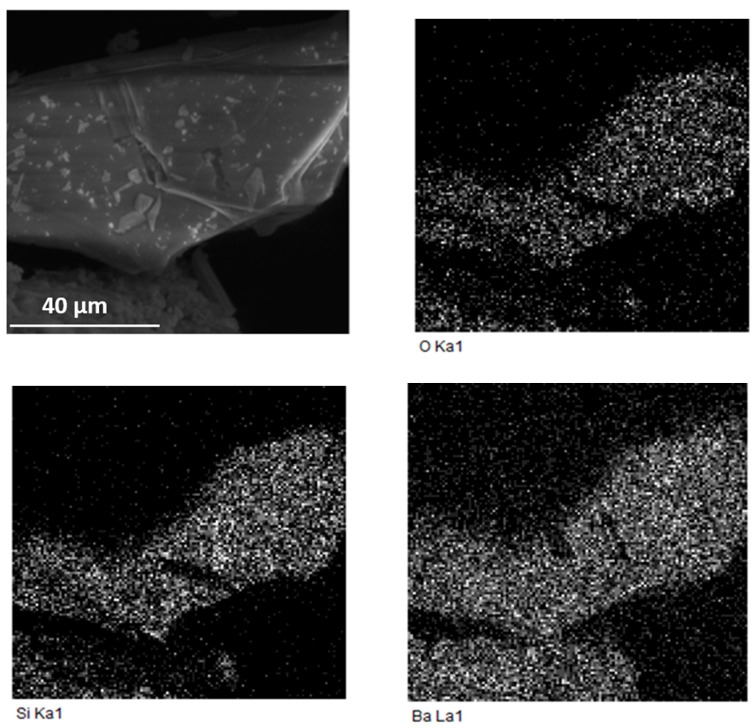

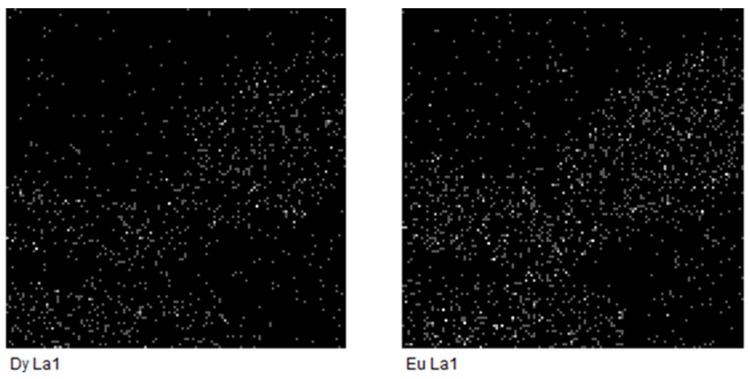
SEM micrographs and relative maps of elements performed by EDS analysis for sample C.

For all samples, we also performed a morphological/compositional evaluation. The morphology was similar in all cases, the agglomerates are well-defined and they are in agreement with the amorphous phase amount. Mapping performed by EDS analysis reveals the presence of the constituent elements Ba, Si, O, Eu and Dy homogeneously distributed over the grain surface. An element mapping was performed as well for the surface and, in the case of sample C (Figure 4), the regular element distributions are evidently compatible with the formation of a doped barium silicate compound.

## 4. Discussion

The purpose of this paper is to improve the properties of Ba_5_Si_8_O_21_:Eu^2+^,Dy^3+^ and to make them comparable to those of other well-studied phosphors, such as strontium aluminates doped with Eu^2+^ and Dy^3+^.

It is well known that Ba_5_Si_8_O_21_:Eu^2+^,Dy^3+^ is an LPP material that can emit sustainable phosphorescence activated by sunlight with a lasting time longer than 16 h. 

Initially, we wanted to improve its LPP characteristics and, for this reason, it was necessary to evaluate the effect of precursors, crucible composition, flux agents, dopants, time and temperatures used for synthesis. 

The results derived from elemental, kinetic, mineralogical and morphological analyses indicate that the variation of these parameters strongly affects the phosphorescence. 

The reference phosphor (sample 1) possesses the following characteristics: Ba/Si = 0.625, Eu/Si = 2.5 × 10^−3^ and Dy/Si = 11.25 × 10^−3^, and it was obtained using BaCO_3_, SiO_2_, Dy_2_O_3_, Eu_2_O_3_ and H_3_BO_3_ at 1250 °C for 10 h in a platinum crucible. The principal phase is Ba_5_Si_8_O_21_ and the phosphor is characterized by *A*_1_ 133 mcd/m^2^.

Table 4 shows that the experimental molar ratios match well with the theoretical values.

Our studies indicate that the best precursors are H_2_SiO_3_ and BaCO_3_ in the molar ratio Ba/Si = 0.7. The suitable dopants are Eu^2+^ and Dy^3+^, and they must be taken in ratio of Eu/Si = 2.8 × 10^−3^ and Dy/Si = 3.6 × 10^−3^. The best flux agent is NaCl, obtained directly from the reaction reported in Section 3.1 between NH_4_Cl and Na_2_SiO_3_. Finally, the optimal thermal treatment conditions are 6 h at 1100 °C and the quartz crucible is the most appropriate. 

Using these synthesis variables, we fabricated the best phosphor (sample C).

**Figure 5:**
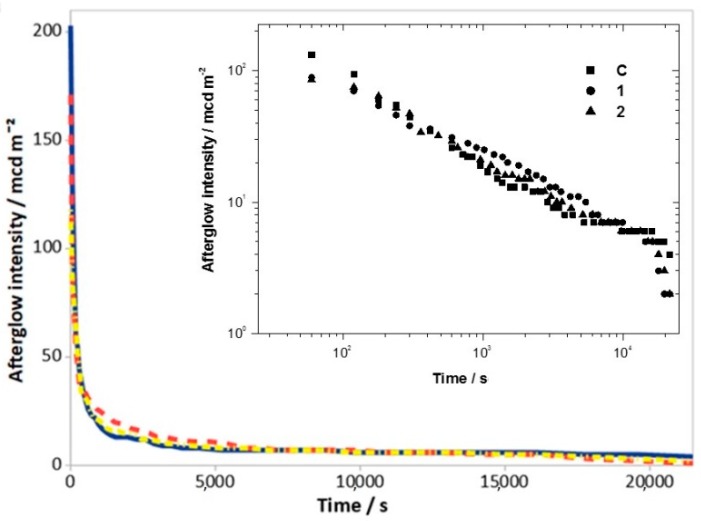
Decay curves of samples C (blue), 1 (red) and 2 (yellow). The upper inset showed the log-log plot.

The reason for this choice comes from the combination of two factors: (i) Ba_5_Si_8_O_21_ as the main crystalline phase and (ii) the best characteristics in terms of luminescence. In fact, compared to sample D (76 wt.% of Ba_5_Si_8_O_21_), even if sample C has a lower amount of Ba_5_Si_8_O_21_ (59% wt.% due to its incomplete crystallization and the preferred orientation of some peaks), its luminescence characteristics are better. Table 2 and Figure 2 indicate a significant difference for the value of the initial emission: *A*_1_ of 180 mcd/m^2^ and 18 mcd/m^2^ for C and D, respectively. Furthermore, its luminescence characteristics are also higher than those of the reference sample (samples 1): *A*_1_ of 180 mcd/m^2^ and 133 mcd/m^2^ for C and 1, respectively (Table 2 and Figure 5). Sample C is better than sample E, which is comparable for the amount of Ba_5_Si_8_O_21_ and for *A*_1_, but the *τ*_2_ value of which is much lower (Table 2). Finally, if we compare sample C with samples F, G, and H, even if we have a similar amount of Ba_5_Si_8_O_21_, they have a lower *A*_1_ value (Table 2).

## 5. Conclusions

In this study, we revealed the possibility to improve the characteristics of known phosphor Ba_5_Si_8_O_21_:Eu^2+^,Dy^3+^. To optimize the process and identify the correct parameters, it was necessary to select an appropriate procedure, as derived by experimental analysis. The variations of precursors, crucible composition, flux agents, dopants, time and temperatures of the treatment strongly influence the LPP characteristics.

Our best synthesis procedure must consider the following parameters:

- quartz crucible that is cheaper and more easily available than the platinum one.
- use of purified coal instead of N_2_/H_2_ as a source of reducing atmosphere, which permits much more disposable and cheaper facilities.
- precursors such as BaCO_3_, Na_2_SiO_3_, NH_4_Cl and an economic flux agent such as NaCl (directly obtained during the synthesis).

Therefore, the data obtained in this study indicate that it is possible to prepare a Ba_5_Si_8_O_21_:Eu^2+^,Dy^3+^ phosphor with improved LPP characteristics: *A*_1_ of 180 mcd/m^2^ respect to 133 mcd/m^2^, which is the reference.

This phosphor was obtained from Ba/Si = 0.7, Eu/Si = 2.8 × 10^−3^, and Dy/Si = 3.6 × 10^−3^, from BaCO_3_, Na_2_SiO_3_, NH_4_Cl, Dy_2_O_3_, and Eu_2_O_3_ at 1100 °C after 6 h of synthesis in a quartz crucible.

## Figures and Tables

**Table 1:** Theoretical molar ratios and synthesis conditions of phosphors.

Sample	Ba/Si	Eu/Si	Dy/Si	Er/Si	Flux Agent	Temperature (°C)	Time (h)
1	0.625	2.5 × 10^−3^	11.25 × 10^−3^	/	H_3_BO_3_	1250	10
2	0.625	2.5 × 10^−3^	11.25 × 10^−3^	/	H_3_BO_3_	1250	10
A	0.700	2.5 × 10^−3^	11.25 × 10^−3^	/	H_3_BO_3_	1100	6
B	0.700	2.5 × 10^−3^	11.25 × 10^−3^	/	NaCl	1100	6
C	0.700	2.8 × 10^−3^	3.6 × 10^−3^	/	NaCl	1100	6
D	0.700	2.8 × 10^−3^	3.6 × 10^−3^	/	H_3_BO_3_	1100	6
E	0.700	2.8 × 10^−3^	/	3.6 × 10^−3^	NaCl	1100	6
F	0.700	2.8 × 10^−3^	3.6 × 10^−3^	/	NaCl	1100	12
G	0.700	2.8 × 10^−3^	3.6 × 10^−3^	/	NaCl	1100	4
H	0.700	2.8 × 10^−3^	3.6 × 10^−3^	/	NaCl	1100	3

**Table 2:** Luminescence kinetic analysis results.

Sample	*A*_1_ (mcd/m^2^)	*t*_1_ (s)	*A*_1_ × *t*_1_ (%)	*A*_2_ (mcd/m^2^)	*t*_2_ (s)	*A*_2_ × *t*_2_ (%)	*t*_m_ (s)
1	133	85	8	35	3759	92	1922
2	94	233	14	20	7056	86	3645
A	94	206	13	13	9602	87	4904
B	109	223	19	23	4431	81	2327
C	180	134	16	20	6358	84	3248
D	18	176	19	3	4393	81	2285
E	213	45	66	10	494	34	269
F	40	225	12	14	4847	88	2536
G	57	186	16	19	3004	84	1595
H	57	151	21	17	1895	79	1023

**Table 3:** QPA (wt.% ± 1) obtained from XRPD analysis.

Phase	A	B	C	D	E	F	G	H
Ba_5_Si_8_O_21_	20	45	59	76	58	57	66	56
Ba_4_Si_6_O_16_	44	15	8	/	7	12	4	10
BaSi_2_O_5_	15	10	13	7	11	11	11	7
Amorphous	21	30	20	17	24	20	19	27

**Table 4:** Theoretical (theo) and experimental (exp) molar ratios for phosphors from elemental analysis.

Sample	Ba/Si (theo)	Ba/Si (exp)	Eu/Si (theo)	Eu/Si (exp)	Dy/Si (theo)	Dy/Si (exp)	Er/Si (theo)	Er/Si (exp)
1	0.625	0.60	2.5 × 10^−3^	2.3 × 10^−3^	11.25 × 10^−3^	10.75 × 10^−3^	/	/
2	0.625	0.59	2.5 × 10^−3^	2.4 × 10^−3^	11.25 × 10^−3^	10.75 × 10^−3^	/	/
A	0.700	0.62	2.5 × 10^−3^	2.6 × 10^−3^	11.25 × 10^−3^	10.45 × 10^−3^	/	/
B	0.700	0.64	2.8 × 10^−3^	2.6 × 10^−3^	11.25 × 10^−3^	11.05 × 10^−3^	/	/
C	0.700	0.63	2.8 × 10^−3^	2.7 × 10^−3^	3.6 × 10^−3^	3.7 × 10^−3^	/	/
D	0.700	0.65	2.8 × 10^−3^	2.7 × 10^−3^	3.6 × 10^−3^	3.5 × 10^−3^	/	/
E	0.700	0.65	2.8 × 10^−3^	2.5 × 10^−3^	/	/	3.6 × 10^−3^	3.4 × 10^−3^
F	0.700	0.66	2.8 × 10^−3^	2.9 × 10^−3^	3.6 × 10^−3^	3.8 × 10^−3^	/	/
G	0.700	0.65	2.8 × 10^−3^	2.7 × 10^−3^	3.6 × 10^−3^	3.5 × 10^−3^	/	/
H	0.700	0.63	2.8 × 10^−3^	2.6 × 10^−3^	3.6 × 10^−3^	3.7 × 10^−3^	/	/

## Supplementary Materials

The following are available online at http://www.mdpi.com/1996-1944/12/1/183/s1, Table S1: Most important peaks of NaCl, BaCO_3_ in the sample C before the heat treatment compared with the reference data; Table S2: Most important peaks of Ba_5_Si_8_O_21_, Ba_4_Si_6_O_16_, BaSi_2_O_5_ compared with the reference data; Figure S1: XRD of sample synthesised with SiO_2_, BaCl_2_ as precursor with molar ratio Ba/Si = 0.625; H_3_BO_3_ as flux agent; Eu_2_O_3_, Dy_2_O_3_ as dopants with molar ratios Eu/Si = 2.8 × 10^−3^ and Dy/Si = 3.6 × 10^−3^. Thermal treatment conditions are 1200 °C for 12 h; Figure S2. XRD of sample synthesised with Na_2_SiO_3_, BaCO_3_ and NH_4_Cl as precursor with molar ratio Ba/Si = 0.7; Eu_2_O_3_, Dy_2_O_3_ as dopants with molar ratios Eu/Si = 2.8 × 10^−3^ and Dy/Si = 3.6 × 10^−3^. Thermal treatment conditions are 1100 °C for 12 h.
