# Supplementary material for: Preparation and Luminescence Properties of Ba5Si8O21 Long Persistent Phosphors Doped with Rare-Earth Elements
Source: Materials (Basel). 2019 Jan 7;12(1):183. doi: 10.3390/ma12010183 (PMC6337357; doi:10.3390/ma12010183)
Supplement: Supplementary file 1 [file materials-12-00183-s001.pdf]

Supplementary Materials: Preparation and Luminescence Properties of $Ba_5Si_8O_{21}$ Long Persistent Phosphors Doped with Rare-Earth Elements

Andrea Silvestri, Maria Laura Ligabue, Gianluca Malavasi and Gigliola Lusvardi *

Table S1. Most important peaks of NaCl, $BaCO_3$ in the sample **C** before the heat treatment compared with the reference data [18].

	**NaCl (theo)**			**NaCl (exp)**		
**d (Å)**	2.81	1.99	3.24	2.81	1.99	3.23
**I (%)**	100	55	10	83	27	6
	**$BaCO_3$ (theo)**			**$BaCO_3$ (exp)**		
**d (Å)**	3.72	3.66	2.62	3.70	3.65	2.62
**I (%)**	100	50	30	100	34	27

Table S2. Most important peaks of $Ba_5Si_8O_{21}$, $Ba_4Si_6O_{16}$, $BaSi_2O_5$ compared with the reference data [18].

		A	B	C	D	E	F	G	H
	**$Ba_5Si_8O_{21}$**								
**d (Ă)**	**3.80**	3.81	3.82	3.84	3.79	3.81	3.84	3.83	3.82
**I (%)**	**100**	37	100	33	51	98	82	100	99
**I(Counts)**	/	1134	1522	1295	2408	2530	1155	2344	3117
**d (Ă)**	**3.27**	3.28	3.28	3.30	3.27	3.27	3.27	3.29	3.29
**I (%)**	**82**	64	69	26	66	100	65	58	47
**I(Counts)**	/	1953	1052	1020	3111	2573	919	1363	1491
**d (Ă)**	**3.73**	3.75	3.75	3.75	3.73	3.73	3.76	3.76	3.74
**I (%)**	**74**	100	55	26	100	99	62	59	86
**I(Counts)**	/	3070	845	1036	4738	2539	867	1387	2730
**d (Ă)**	**6.88**	6.85	6.85	6.88	6.88	6.89	6.88	6.86	6.85
**I (%)**	**13**	12	14	100	13	71	100	77	33
**I(Counts)**	/	380	447	3925	628	2010	1406	1810	1038
	**$Ba_4Si_6O_{16}$**								
**d (Ă)**	**3.74**	3.75	3.78	3.75	3.73	3.73	3.76	3.76	3.74
**I (%)**	**100**	100	84	26	100	99	62	59	86
**I(Counts)**	/	3070	1274	1036	4738	2539	867	1387	2730
**d (Ă)**	**3.67**	3.68	/	/	/	/	/	/	/
**I (%)**	**51**	80	/	/	/	/	/	/	/
**I(Counts)**	/	2452	/	/	/	/	/	/	/
**d (Ă)**	**3.25**	3.25	3.25	3.26	/	3.22	3.25	3.24	3.24
**I (%)**	**45**	52	55	24	/	68	72	43	59
**I(Counts)**	/	1601	1504	932	/	3065	1010	1012	1860
**d (Ă)**	**3.29**	3.28	3.28	3.30	3.27	3.27	3.27	3.29	3.29
**I (%)**	**41**	64	69	26	66	100	65	58	47
**I(Counts)**	/	1953	1052	1020	3111	2573	919	1363	1491

	**$BaSi_2O_5$**								
**d (Ă)**	**3.10**	3.10	3.13	3.12	3.11	3.10	3.12	3.12	3.11
**I (%)**	**100**	43	59	21	21	43	53	47	100
**I(Counts)**	/	1318	900	811	995	1108	748	1094	3162
**d (Ă)**	**3.97**	3.98	4.02	4.00	3.96	3.97	4.02	3.99	3.99
**I (%)**	**85**	58	53	30	9	63	28	13	48
**I(Counts)**	/	1779	807	1169	415	1628	388	297	1521
**d (Ă)**	**3.34**	3.35	3.36	3.36	3.34	3.35	3.36	3.36	3.36
**I (%)**	**70**	48	31	14	9	38	34	30	23
**I(Counts)**	/	1470	479	538	434	988	478	703	718
**d (Ă)**	**3.42**	3.43	3.44	3.41	3.42	3.43	3.41	3.44	3.44
**I (%)**	**50**	34	25	36	3	56	20	14	19
**I(Counts)**	/	1054	758	1416	157	2045	279	330	616

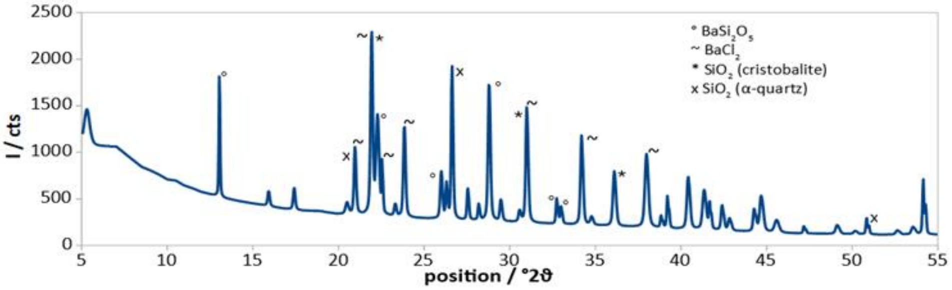

Figure S1. XRD of sample synthesised with SiO_2, $BaCl_2$ as precursor with molar ratio Ba/Si = 0.625; H_3BO_3 as flux agent; Eu_2O_3, Dy_2O_3 as dopants with molar ratios Eu/Si = 2.8×10^{-3} and Dy/Si = 3.6×10^{-3}. Thermal treatment conditions are 1200 °C for 12 h. The crucible is in alumina.

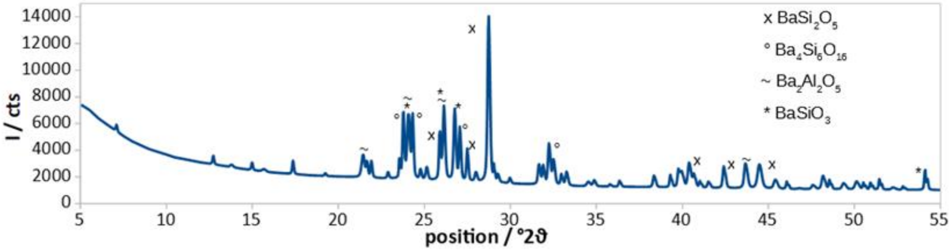

Figure S2. XRD of sample synthesised with Na_2SiO_3, $BaCO_3$ and NH_4Cl as precursor with molar ratio Ba/Si = 0.7; Eu_2O_3, Dy_2O_3 as dopants with molar ratios Eu/Si = 2.8×10^{-3} and Dy/Si = 3.6×10^{-3}. Thermal treatment conditions are 1100 °C for 12 h. The crucible is in alumina.
